# Preparation of a (Ca,Sr,Ba)ZrO_3_ Crucible by Slip Casting for the Vacuum Induction Melting of NiTi Alloy

**DOI:** 10.3390/ma17081924

**Published:** 2024-04-22

**Authors:** Shijia Ding, Mingliang Li, Hailong Wang, Jinpeng Zhu, Gang Shao, Hongliang Xu, Hongxia Lu, Rui Zhang

**Affiliations:** 1 School of Materials Science and Engineering, Zhengzhou University, Zhengzhou 450001, China; 2 Zhongyuan Critical Metals Laboratory, Zhengzhou University, Zhengzhou 450001, China

**Keywords:** (Ca,Sr,Ba)ZrO_3_, NiTi alloy, corrosion resistance, slip casting

## Abstract

Vacuum induction melting is a more energy-efficient process for the preparation of a titanium alloy with good homogeneity and low cost. But the crucial problem for this technology is in developing a crucible refractory with high stability. In the present work, a novel (Ca,Sr,Ba)ZrO_3_ crucible was prepared by slip casting and its performance in melting NiTi alloy was studied. The results showed that a single solid solution was formed with a homogeneous distribution of metal elements after sintering at 1500 °C. It was found that the total content of oxygen and nitrogen remaining in the TiNi alloy after melting in the (Ca,Sr,Ba)ZrO_3_ crucible was 0.0173 wt.%, which fulfills the ASTM standard on biomedical TiNi alloys. The good resistance of the (Ca,Sr,Ba)ZrO_3_ crucible to molten NiTi has a relationship with the sluggish diffusion effect of high-entropy ceramics. This study provides insights into the process of designing highly suitable crucible material for melting a NiTi alloy.

## 1. Introduction

As a shape memory alloy (SMA), a NiTi alloy with good functional properties and high mechanical strength has been widely applied in aerospace, robotics and biomedical fields [1,2]. The shape-memory effect of the alloys is obtained by the diffusionless solid phase transition between austenitic and martensitic structures [3]. The martensite start temperature of NiTi is correspondingly reduced with the increase in the atom ratio of Ni/Ti [3,4]; for example, the phase transition temperature drops by 10 K for every 0.1 atomic percent increases in Ni. Therefore, the content of the impurity elements must be strictly controlled in the melting process. Compared to vacuum-consumable arc re-melting (VAR), the molten NiTi alloy with higher superheat and fluidity can be obtained by vacuum induction melting (VIM) without disturbance from the water-cooled copper crucible. VIM is an energy-saving method for producing NiTi alloy with good composition uniformity.

Due to their high chemical activity, titanium alloys can easily react with crucible materials at high temperatures. Nitrides [5], carbides [6]**,** and oxide crucibles [7,8,9,10] have been studied for crucible materials. Kartavykh et al. reported that the BN crucible could react with the titanium melt to form Ti borides. The thickness of the reaction layer was about 250 μm [5]. There was also an obvious reaction layer between the graphite crucible and titanium alloy after melting [6]. Based on the thermodynamic calculation, Kostov et al. found that Al_2_O_3_, ZrO_2_, CaO, and Y_2_O_3_ could be used as crucible materials for melting titanium alloys [11]. However, an interaction zone between Al_2_O_3_ and titanium alloy was observed [10]. ZrO_2−x_ and α-Ti(O) were detected after melting with the ZrO_2_ crucible [12]. The thermal shock resistance of the Y_2_O_3_ crucible was poor [13]. The application of the CaO crucible was affected by its hygroscopic nature [8].

Alkaline earth zirconates with high chemical stability belong to the perovskite structure. Song et al. reported that the thickness of the interface between the CaZrO_3_ crucible and Ti-Cu alloy was about 740 μm [14]. Meng et al. investigated the corrosion resistance of the Y_2_O_3_-doped SrZrO_3_ crucible for melting NiTi alloy. The experiment results showed that no obvious reaction layer between the crucible and the NiTi alloy was found [15]. The thickness of the reaction layer between the BaZrO_3_ crucible and the Ni_2_Ti alloy was about 270 μm [16]. To utilize vacuum induction melting for titanium alloys, crucible materials with high stability and no reaction with titanium alloys at high temperatures are required.

The view of “high-entropy ceramics” was developed from the concept of a high-entropy alloy. In the last ten years, there has been growing attention on high-entropy ceramics because of their controlled coefficient of thermal expansion, dielectric constant, and other properties [17]. Compared to single-component materials, high-entropy ceramics have superior stability and corrosion resistance due to the high-entropy, lattice distortion, sluggish diffusion, and cocktail effects. According to the corrosion resistance of zirconates, a novel entropy stable (Ca,Sr,Ba)ZrO_3_ ceramic was designed and obtained by our group [18]. However, it has not yet been formed into a crucible and studied under VIM.

Slip casting is a technique that uses the water absorption of gypsum molds to inject the prepared slurry into the mold [19,20,21]. After the moisture has been absorbed, it forms a green body with a certain strength. The advantages of this method are its low cost and easy preparation of large-sized and complex-shaped products.

Therefore, CaCO_3_, SrCO_3_, BaCO_3_, and ZrO_2_ powders were used as raw materials to prepare (Ca,Sr,Ba)ZrO_3_ powder through the solid state reaction. The effect of different solid contents in the slurry for the (Ca,Sr,Ba)ZrO_3_ crucible was also investigated. Subsequently, the interface between the NiTi alloy and the crucible was examined after vacuum induction melting. The content of impurities in the NiTi alloy after melting was analyzed and the feasibility of the (Ca,Sr,Ba)ZrO_3_ crucible for melting the NiTi alloy was discussed.

## 2. Materials and Methods

### 2.1. Preparation of the (Ca,Sr,Ba)ZrO_3_ Crucible

**Figure 1:**
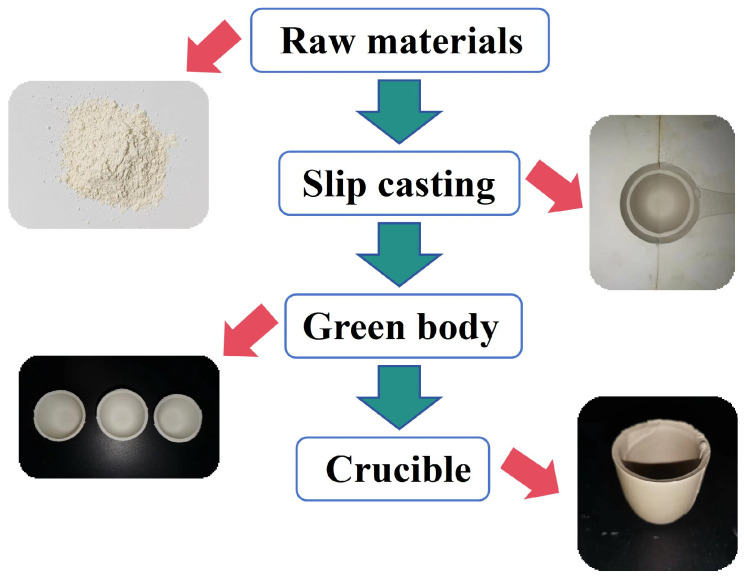
The flowchart of the preparation of crucibles.

CaCO_3_, SrCO_3_, BaCO_3_ (99.9% purity, 1–2 μm, HWRK Chem. Co., Ltd., Beijing, China), and ZrO_2_ (99.99% purity, 1–3 μm, HWRK Chem. Co., Ltd., Beijing, China) powders were mixed in the molar ratio of 1:1:1:3. After milling for 8 h with ZrO_2_ balls, the slurry was dried at 50 °C. Then, the mixed powders were heated at 1500 °C for 4 h to synthesize (Ca,Sr,Ba)ZrO_3_. For the preparation of the slurry used in slip casting, the obtained (Ca,Sr,Ba)ZrO_3_ powder was mixed with distilled water and gum Arabic. The content of the powder and gum Arabic was 76–80 wt.% and 1.2–1.0 wt.%, respectively. The slurry was defoamed in a vacuum defoamer for 15 min. In slip casting, the remaining slurry was poured out when the wall thickness of the crucible was about 3~5 mm. The dried green body was obtained at 25 °C for 48 h. Finally, the (Ca,Sr,Ba)ZrO_3_ crucible was produced by sintering at 1600 °C for 6 h. The flowchart of the preparation of crucibles is shown in Figure 1.

### 2.2. Melting Experiment

The NiTi alloy was melted using the (Ca,Sr,Ba)ZrO_3_ crucible in a vacuum induction furnace (VIF10-17, SGM, Luoyang, China). The vacuum induction furnace was purged twice with high-purity argon gas when the vacuum degree dropped to below 10^−2^ Pa. The melting experiment was carried out when the argon gas backfilled to 5000 Pa. The melting temperature was increased from 30 °C to 1200 °C in 30 min, followed by an increase to 1500 °C in 20 min. Finally, the crucible was cooled to room temperature in the furnace after being held at 1500 °C for 15 min.

### 2.3. Analysis Method

The phase composition of the (Ca,Sr,Ba)ZrO_3_ powders was characterized by X-ray diffraction analysis (XRD, PANalytical EMPYRAN, Malvern Panalytical Ltd., Malvern, UK). The microstructure and element distribution of the sintered samples was analyzed by scanning electron microscopy (FESEM, JSEM-6700F, JEOL, Tokyo, Japen). The laser particle size analyzer (Mastersizer 3000, Malvern, UK) was used for measuring the particle size of the milled powder. The viscosity of the (Ca,Sr,Ba)ZrO_3_ slurry was tested using a digital viscometer (Shanghai Pingxuan Scientific Instrument Co., Ltd., Shanghai, China). The density of the (Ca,Sr,Ba)ZrO_3_ crucible was obtained using the Archimedes method. The polished interface between the NiTi alloy and the (Ca,Sr,Ba)ZrO_3_ crucible were characterized by SEM. The content of oxygen and nitrogen in the NiTi alloy was tested via a nitrogen-oxygen analyzer (EMGA-830, HORIBA, Kyoto, Japan). The other chemical compositions were detected using the Inductive Coupled Plasma Emission Spectrometer (Agilent 5110, Santa Clara, CA, USA).

## 3. Results and Discussion

**Figure 2:**
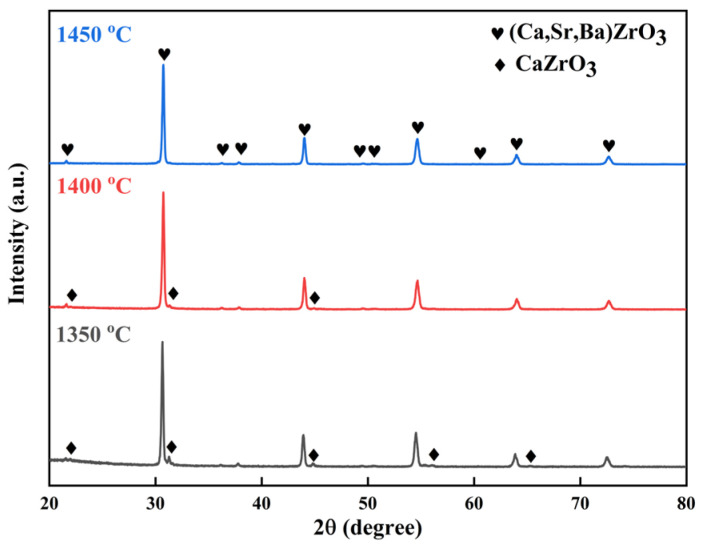
XRD patterns of (Ca,Sr,Ba)ZrO_3_ prepared at different sintering temperatures.

The XRD patterns of the (Ca,Sr,Ba)ZrO_3_ powders synthesized at different temperatures are shown in Figure 2. It can be seen that the main phase of the samples synthesized at 1350 °C and 1400 °C was (Ca,Sr,Ba)ZrO_3_. However, there were also some small peaks of CaZrO_3_ in the samples. With the increase in the sintering temperature, only the peaks of (Ca,Sr,Ba)ZrO_3_ could be observed at 1450 °C, indicating that a single solid solution (Ca,Sr,Ba)ZrO_3_ with tetragonal perovskite structure had formed. The preparation temperature of (Ca,Sr,Ba)ZrO_3_ was at least 1450 °C.

**Figure 3:**
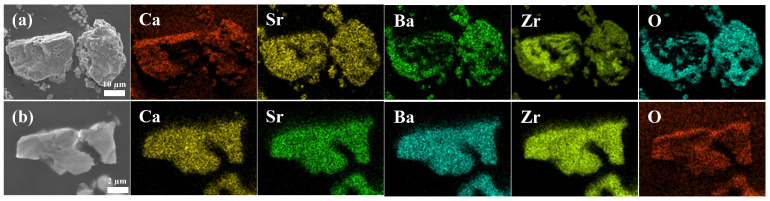
SEM micrographs of the powder and the corresponding EDS mappings of (Ca,Sr,Ba)ZrO_3_ sintered at (**a**) 1450 °C and (**b**) 1500 °C.

Figure 3 displays SEM images and corresponding EDS mappings of the samples synthesized at different temperatures. As shown in these images, the distribution of Ca, Sr and Ba cations became gradually homogeneous at the micrometer level with the increase in sintering temperature. For the (Ca,Sr,Ba)ZrO_3_ powder obtained at 1500 °C (Figure 3b), Ca, Sr, and Ba was distributed homogeneously and randomly without segregation at the micrometer level, which was an indication that a single-phase and homogenous solid solution (Ca,Sr,Ba)ZrO_3_ had been formed at micrometer level. Therefore, 1500 °C was selected as the preparation temperature of the (Ca,Sr,Ba)ZrO_3_.

**Figure 4:**
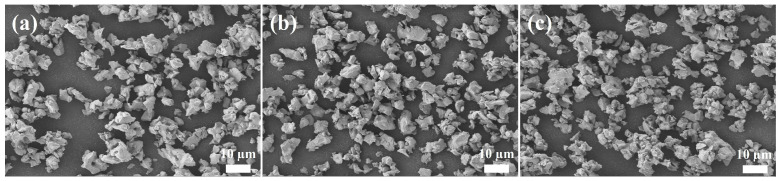
SEM micrographs of the powder after milling: (**a**) 6 h, (**b**) 8 h, and (**c**) 10 h.

To attain a high density and favorable mechanical properties of the crucible, it is crucial to maintain a low viscosity of the slurry during slip casting [22]. The property of the slurry is influenced by several factors, such as the type of dispersant, powder size, and solid content. The polymer chains of gum Arabic play an electrostatic stabilizing role by adsorbing on to the surface of the powder, which reduces the viscosity of slurry and prevents particle agglomeration. It is also important to note that the size of the powder has a significant effect on slip casting. The SEM micrographs of the milled powder are shown in Figure 4a–c. It can be seen that the milled powders had an irregular shape.

Table 1 shows the average particle size of the (Ca,Sr,Ba)ZrO_3_ powders after being milled for different amounts of time. With the increase in the milling time, the average particle sizes of the powders first decreased and then increased. The average particle sizes of the powders milled for 6 h, 8 h, and 10 h were 1.535 µm, 1.521 µm, and 1.652 µm, respectively. The D_10_ and D_50_ of the powder milled for 8 h were 0.650 µm and 2.054 µm, respectively. The bigger average particle size of the powder milled for 10 h may be related to the larger surface energy of the smaller particles, which lead to the agglomeration. The settling velocity *V* of particles in suspension was calculated by Stokes’ formula equation:

(1) $$V=\frac{2}{9}r^{2}\left(\frac{\rho_{p}-\rho_{w}}{\eta }\right)g$$

where *r* is the average particle radius, $\rho_{p}$ is particle density, $\rho_{w}$ is the density of the suspension, g is gravitational acceleration, and *η* is the viscosity of slurry. According to Equation (1) [23], a smaller average particle size could reduce the settling velocity of (Ca,Sr,Ba)ZrO_3_ particles and promote the stable dispersion of (Ca,Sr,Ba)ZrO_3_ particles. Smaller particles also possessed a larger surface area, resulting in a greater sintering drive force during the subsequent sintering process [24]. Therefore, the powder milled for 8 h was used to prepare the (Ca,Sr,Ba)ZrO_3_ slurry for slip casting.

**Figure 5:**
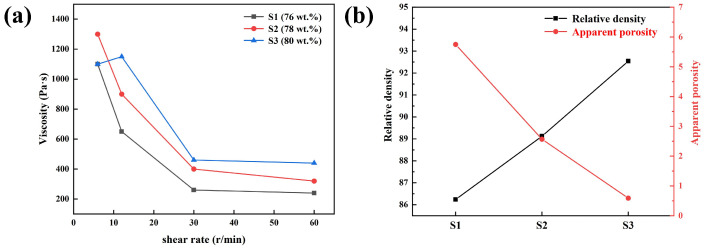
(**a**) The viscosity curves of the slurry in different formulations; (**b**) the relative density and apparent porosity curves of the (Ca,Sr,Ba)ZrO_3_ crucible with different formulations.

The bond strength of the green body after slip casting is primarily influenced by the solid content of the slurry. If the solid content is too low, the green body may crack because the binding force is insufficient to resist shrinkage stress. Conversely, if the solid content is too high, the low permeability of the slurry may lead to a large humidity gradient in the thickness direction of the green body. This hinders the ion exchange of the slurry and gypsum mold, which produce different shrinkage stresses to result in the crack of the green body. The solid and gum Arabic contents in the different formulations are shown in Table 2. From the table, there were three different formulations with solid contents of the slurry of 76 wt.%, 78 wt.%, and 80 wt.%. The corresponding samples were named S1, S2, and S3, respectively. Figure 5a illustrates the relationship between the solid content and the viscosity of the slurry. The viscosity of the slurry decreased with the shear rate increase, demonstrating a non-Newtonian shear-thinning property, as shown in Figure 5a [20]. It can be seen that the viscosity of S3 is higher than S1 and S2. The viscosity of the slurry increased slowly as the solid content increased. A possible explanation for this was that the number of gum Arabic molecules per powder particle decreased as the solid content increased when the amount of dispersant was certain in the slurry.

The double electron shell structure among the particles weakened, resulting in a weakened spatial steric effect and a gradual increase in the viscosity of the slurry. In addition, the increase in the viscosity of the slurry may be related to the distance between the particles in the slurry shortening and the organic molecules adsorbed on their surface overlapping [25]. The fluidity of the slurry with the higher solid content was too low to cast. The slurry with the low solid content had good flowability, but the adsorption rate of the gypsum mold was faster during slip casting. This resulted in an uneven surface of the green body of the (Ca,Sr,Ba)ZrO_3_ crucible. Generally, slip casting can be carried out when the viscosity of the slurry is below 1000 mpa·s. When the glass rod is lifted out of the slurry, continuous filaments will be formed when the slurry flows down, which is conducive to the formation of the green body on the inner wall of the gypsum.

Figure 5b shows the relative density and apparent porosity curves of the crucibles with different formulations. The relative density of the (Ca,Sr,Ba)ZrO_3_ crucibles increased with the increase in the solid content. The maximum relative density was 92.5% for S3 with the solid content of the slurry of 80 wt.%. Meanwhile, the corresponding apparent porosity of the crucibles decreased. A higher density of the crucible can effectively reduce the erosion of the titanium alloy’s liquid on the surface of the crucible [26]. Therefore, the (Ca,Sr,Ba)ZrO_3_ slurry was prepared with 1.0 wt.% gum Arabic solution and 80 wt.% (Ca,Sr,Ba)ZrO_3_ powder in this study.

**Figure 6:**
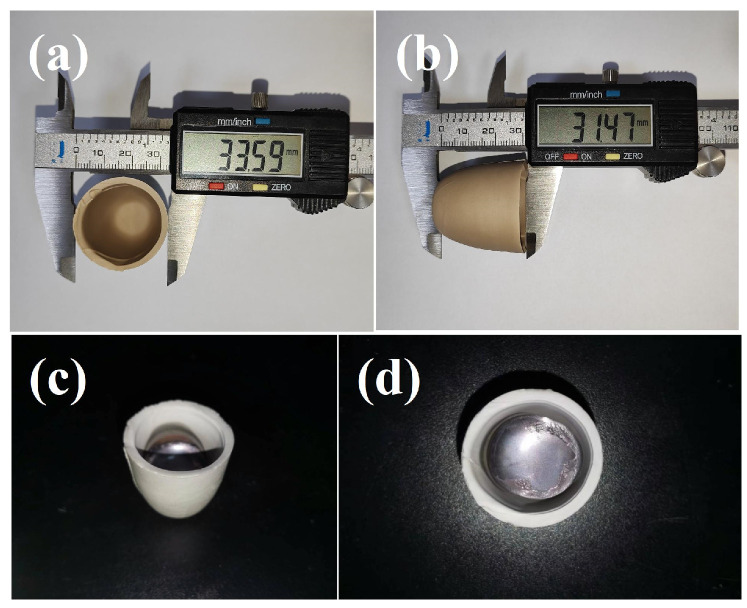
External surface of the crucible after sintering (**a**,**b**), macrograph of the NiTi alloy melted in the (Ca,Sr,Ba)ZrO_3_ crucible (**c**,**d**).

As shown in Figure 6a,b, the height and external diameter of the sintered crucible were about 31.47 mm and 33.59 mm, respectively. According to these images, the outer surface of the crucible was smooth. The inner surface of the crucible was free of obvious defects, such as pores and pinholes. The macroscopic surface of the crucible after melting is displayed in Figure 6c,d. It can bee seen that there were no obvious cracks in the crucible after melting in a vacuum induction furnace, indicating that the crucible had good thermal shock resistance. After cooling to room temperature, the surface of the titanium alloy was flat and pure, indicating that the (Ca,Sr,Ba)ZrO_3_ crucible had good corrosion resistance for the melting of the titanium alloy [27].

**Figure 7:**
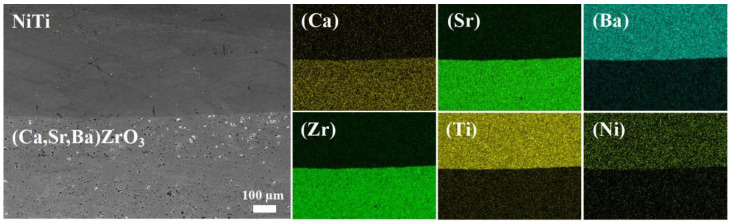
SEM-EDS analysis of the interface between the NiTi alloy and (Ca,Sr,Ba)ZrO_3_ crucible.

**Figure 8:**
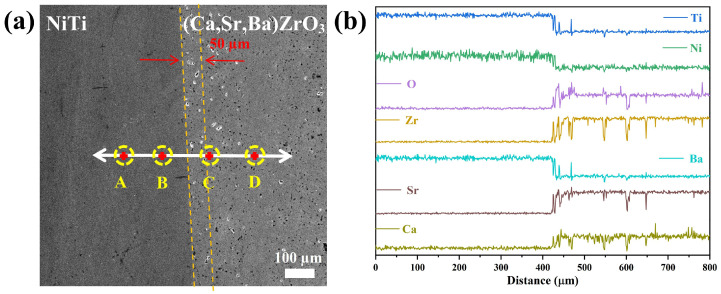
(**a**) SEM image of the interface between the NiTi alloy and (Ca,Sr,Ba)ZrO_3_ crucible, and the corresponding (A–D) point scanning location; (**b**) the line scanning result of the white line in the SEM image.

Figure 7 shows the SEM micrographs and relevant mapping results of the interface between the NiTi alloy and the (Ca,Sr,Ba)ZrO_3_ crucible after melting in VIM. It can be seen that the interface between the NiTi and (Ca,Sr,Ba)ZrO_3_ was very clear. If there were some small pores on the surface of the crucible, then the infiltration of the melted titanium alloy may have happened due to the capillary force caused by the pores [26]. In addition, the presence of microcracks in the crucible also promoted the penetration of the alloy melt into the ceramic. From the distribution of elements, Ca, Sr, and Zr elements were concentrated on the side of the crucible. There was no sign of diffusion into the NiTi alloy side. It is worth noting that Ba was detected on the side of the NiTi alloy, which was ascribed to the precision limit of EDS line scanning. EDS mapping failed to distinguish between Ba and Ti. As the matter of fact, the content of Ba in the NiTi alloy was almost zero, according to the EDS results of points A and B in Table 3. Table 3 shows the corresponding EDS composition of points A–D in Figure 8a. The atomic ratio of Ni and Ti at points A and B in the alloy side was close to 1:1, which benefitted the shape memory property of the NiTi alloy [3,28]. Meanwhile, the result of points C and D in the (Ca,Sr,Ba)ZrO_3_ side also basically matched the chemical composition of the crucible.

Figure 8a shows the SEM micrographs and corresponding (A~D) point scanning locations. Based on the line scanning and point scanning results of the interface in Figure 8a, the alloy side was on the left side and the crucible was on the right side. The consequence of the EDS line scanning from the NiTi alloy to the crucible is shown in Figure 8b. There was no noticeable change in the content of Ca, Sr, Zr, and O in the NiTi alloy. Some variations in the curves of each element in the (Ca,Sr,Ba)ZrO_3_ side were attributed to the penetration of the melted NiTi alloy. From the alloy side to the crucible side, the elements underwent a sharp change at the interface between the NiTi alloy and (Ca,Sr,Ba)ZrO_3_ crucible. The elements in the alloy side decreased rapidly, while the corresponding elements on the crucible side increased slowly. According to the range of the change in the elements, the thickness of the interface between the NiTi alloy and the (Ca,Sr,Ba)ZrO_3_ crucible was about 50 µm.

Table 4 displays the content of impurities in NiTi alloys after VIM with the (Ca,Sr,Ba)ZrO_3_ crucible and other crucibles from the literature [26,29]. After VIM with the (Ca,Sr,Ba)ZrO_3_ crucible, the total content of oxygen and nitrogen was 0.0173 wt.%, which was lower than the corresponding content of the BaZrO_3_ crucible. The values also met with the ASTM F2063-05 standard [29] below 0.05 wt.%. Furthermore, because of the low content of Ca, Sr, and Ba in the alloy, it can be deduced that there was no obvious reaction between the NiTi alloy and the (Ca,Sr,Ba)ZrO_3_ crucible during VIM. It should be noted that the alloy contained a relatively high amount of Zr compared to other elements, which may be due to the substitution of Zr into the Ti lattice in the solid solution of the titanium-rich binary alloy.

**Figure 9:**
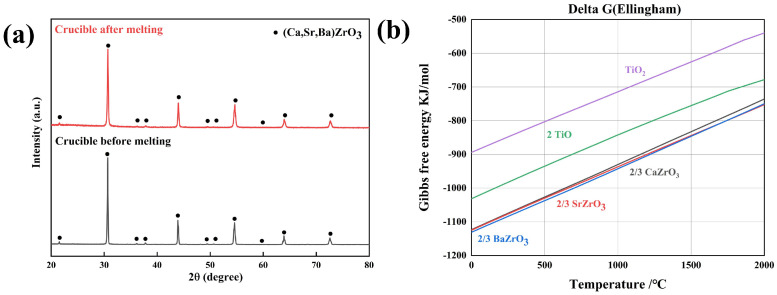
(**a**) XRD patterns of the surface of the (Ca,Sr,Ba)ZrO_3_ crucible before and after melting NiTi alloy; (**b**) the Gibbs free energy for the formation of TiO_2_, TiO, CaZrO_3_, SrZrO_3_, and BaZrO_3_.

As displayed in Figure 9a, there was no new phase on the surface of the (Ca,Sr,Ba)ZrO_3_ crucible after melting the NiTi alloy. It was revealed that (Ca,Sr,Ba)ZrO_3_ is a promising crucible material for the melting of titanium alloys. Figure 9b illustrates the dependence of Gibbs free energy on the temperature of TiO_2_, TiO, CaZrO_3_, SrZrO_3_, and BaZrO_3_ obtained using HSC software (version 6.0). From Figure 9b, the Gibbs free energy of CaZrO_3_, SrZrO_3_, and BaZrO_3_ is lower than that of titanium oxides (TiO_2_ and TiO). From the perspective of mixed entropy, high-entropy (Ca,Sr,Ba)ZrO_3_ ceramics should have a lower Gibbs free energy than their individual components, and this suggests that the (Ca,Sr,Ba)ZrO_3_ crucible exhibits good high-temperature chemical stability and does not react with the alloy melt. Physical erosion may have appeared due to the challenges of interface reaction [12]. It occurs when the crucible is melted by the molten metal, causing the melt to enter the inside of the crucible through capillary forces. This results in the particles in the outer layer of the (Ca,Sr,Ba)ZrO_3_ crucible dissolving and coming out, which promotes the further penetration of the melt into the crucible.

**Figure 10:**
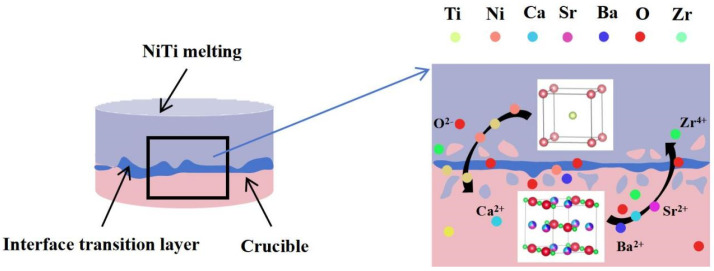
Schematic diagram of physical erosion between the NiTi alloy and (Ca,Sr,Ba)ZO_3_ crucible.

The interactional schematic diagram between the NiTi alloy melt and the (Ca,Sr,Ba)ZrO_3_ crucible is shown in Figure 10. The touch position of the (Ca,Sr,Ba)ZrO_3_ crucible softened when it came into contact with the molten NiTi alloy at 1500 °C, resulting in the gradual dissolution of the crucible grains into the molten NiTi. The ions released, such as Zr^4+^ and O^2−^, diffused in the NiTi alloy melt. The sluggish diffusion effect [30] in the high-entropy (Ca,Sr,Ba)ZrO_3_ crucible effectively prevents the further diffusion of the elements on both sides of the interface layer. This is because the elements of Ca, Sr, and Ba in the (Ca,Sr,Ba)ZrO_3_ structure occupied the same lattice position uniformly. And the atoms at adjacent points in each lattice position were different. As a result, the local energy of each atom occupying the lattice point position was also different. In the (Ca,Sr,Ba)ZrO_3_, when a metal atom diffused to a low energy position, it was likely to become trapped and unable to escape. Conversely, if a metal atom diffused to a high-energy position, it had a higher chance of returning to its original position. These factors contribute to a slower diffusion of the metal element into the alloy melt. When atoms in the alloy melt attempt to diffuse into the (Ca,Sr,Ba)ZrO_3_ structure, the same situation occurs. The combination of the above analysis suggests that the (Ca,Sr,Ba)ZrO_3_ crucible had significantly better resistance to NiTi alloy melt erosion.

## 4. Conclusions

In the present work, a (Ca,Sr,Ba)ZrO_3_ crucible was designed and prepared by slip casting. The feasibility of the (Ca,Sr,Ba)ZrO_3_ crucible for melting the NiTi alloy was discussed. The following conclusions have been summarized:

(1) X-ray diffraction and scanning electron microscopy analyses collectively indicated that a single solid solution was formed with a homogeneous distribution of metal elements after sintering at 1500 °C.
(2) There was no new phase on the surface of the (Ca,Sr,Ba)ZrO_3_ crucible after melting the NiTi alloy, and the interface between NiTi and (Ca,Sr,Ba)ZrO_3_ was very clear. Most importantly, the total content of oxygen and nitrogen elements was 0.0173 wt.% after vacuum induction melting, which was beneficial for the performance of the titanium alloy.
(3) The good resistance of the (Ca,Sr,Ba)ZrO_3_ crucible to molten NiTi has a relationship with the sluggish diffusion effect of high-entropy ceramics. The combination of the properties indicates that (Ca,Sr,Ba)ZrO_3_ is a potential crucible material for high-reactivity titanium alloys through vacuum induction melting.

## Figures and Tables

**Table 1:** The powder diameter was measured at various milling times.

Grinding Time (h)	Average Particle Size (µm)	D_10_ (µm)	D_50_ (µm)
6	1.535	0.658	2.089
8	1.521	0.650	2.054
10	1.621	0.696	2.399

**Table 2:** The solid and gum Arabic content in the different formulations.

Sample	Solid Content of Slurry (wt.%)	Gum Arabic Content (wt.%)
S1	76	1.2
S2	78	1.1
S3	80	1.0

**Table 3:** EDS results of points A–D in Figure 8a.

Point	Ca (at.%)	Sr (at.%)	Ba (at.%)	Zr (at.%)	O (at.%)	Ti (at.%)	Ni (at.%)	Possible Phase
A	-	-	-	-	-	52.43	47.57	NiTi
B	-	-	-	-	-	50.91	49.09	NiTi
C	6.80	6.65	7.23	23.54	55.77	-	-	(Ca,Sr,Ba)ZrO_3_
D	6.64	6.91	7.83	23.67	54.95	-	-	(Ca,Sr,Ba)ZrO_3_

**Table 4:** The content of the impurities in the NiTi alloy after vacuum induction melting.

Crucible	The Content of Elements [wt.%]					
	O	N	Ca	Sr	Ba	Zr
(Ca,Sr,Ba)ZrO_3_	0.0170	0.0003	0.0018	0.00006	0.0001	0.0785
BaZrO_3_ [29]	0.0370	0.00014	-	-	0.00007	0.033
ZrO_2_ [26]	1.046	-	-	-	-	0.007
Al_2_O_3_ [26]	1.600	-	-	-	-	-

## Data Availability

Data are contained within the article.
